# In-Depth Qualitative Analysis of Lime Essential Oils Using the Off-Line Combination of Normal Phase High Performance Liquid Chromatography and Comprehensive Two-Dimensional Gas Chromatography-Quadrupole Mass Spectrometry

**DOI:** 10.3390/foods8110580

**Published:** 2019-11-16

**Authors:** Mariosimone Zoccali, Barbara Giocastro, Ivana L. Bonaccorsi, Alessandra Trozzi, Peter Q. Tranchida, Luigi Mondello

**Affiliations:** 1 Department of Mathematical and Computer Science, Physical Sciences and Earth Sciences, University of Messina, 98166 Messina, Italy; 2 Department of Chemical, Biological, Pharmaceutical and Environmental Sciences, University of Messina, 98168 Messina, Italy; bgiocastro@unime.it (B.G.); ivabonaccorsi@unime.it (I.L.B.); trozzia@unime.it (A.T.); lmondello@unime.it (L.M.); 3 Chromaleont s.r.l., c/o Department of Chemical, Biological, Pharmaceutical and Environmental Sciences, University of Messina, 98168 Messina, Italy; 4 Unit of Food Science and Nutrition, Department of Medicine, University Campus Bio-Medico of Rome, 00128 Rome, Italy; 5 BeSep s.r.l., c/o Department of Chemical, Biological, Pharmaceutical and Environmental Sciences, University of Messina, 98168 Messina, Italy

**Keywords:** comprehensive two-dimensional gas chromatography, essential oil, quadrupole mass spectrometry, citrus essential oil, lime essential oil

## Abstract

The present research is focused on the in-depth qualitative analysis of three types of lime essential oil (EO), viz., Key (A and B) and Persian, using the off-line combination of normal phase high performance liquid chromatography (NP-HPLC) and comprehensive two-dimensional gas chromatography–quadrupole mass spectrometry (GC × GC-QMS). The first analytical dimension (NP-HPLC) was exploited for the isolation of the hydrocarbon constituents from the oxygenated ones. Each fraction was then reduced in volume and analyzed using (cryogenic modulation) GC × GC-QMS. Peak assignment was carried out through the combined use of mass spectral database and linear retention index matching processes. The powerful four-dimensional technology enabled the separation and identification of a very high number (153) of lime essential oil volatile compounds.

## 1. Introduction

Two varieties of sour lime, namely Key or Mexican (*Citrus aurantifolia* Swingle) and Persian (*Citrus latifolia* Tanaka), find wide use in the flavor industry. Distilled Key lime oil is the most common product, with its aroma deriving from transformation processes (hydration, elimination, rearrangement reactions) which occur during the distillation process. Cold-pressed lime oil is characterized by a fragrant citrus aroma and is used in perfumery, as well as in the flavor industry. Different types of cold-pressing processes provide different types of lime oils: (I) a screw press is used to attain a juice–oil–pulp mixture, followed by centrifugation to isolate the essential oil. Such a procedure is used only for Key limes and yields the type A oil; (II) the peel is subjected to gentle grating, with the oil washed away through the application of water. After, the oil is recuperated through centrifugation. Such a process is applied to both Key (type B oil) and Persian limes [1].

The volatile fraction of lime oils is lower than other cold-pressed citrus oils (e.g., 85% against 99% of sweet orange oil), and is composed of a variety of mono- and sesquiterpenes (both hydrocarbons and oxygenated), along with aliphatic alkanes, alcohols, and aldehydes [2].

Gas chromatography–mass spectrometry (GC-MS) is certainly the prime analytical choice for the qualitative untargeted analysis of the volatile fraction of lime essential oil; identification is often achieved through MS database matching, the use of linear retention index (LRI) information, and the co-injection of pure standard compounds. The GC-MS analysis is commonly performed using a conventional (i.e., 30 m × 0.25 mm ID × 0.25 μm d_f_) low-polarity column and a unit-mass resolution mass spectrometer [2,3].

Even though the utility of GC-MS is not herein doubted, it has been previously shown that the on-line combination of normal phase liquid chromatography (NP-LC), and GC-MS is of high analytical usefulness within the context of lime essential oil analysis, and more in general in that of essential oils. The NP-LC process achieves a polarity-based separation, thus isolating the hydrocarbons from the oxygenated compounds. After each fraction is subjected to a GC-MS analysis, reducing the chance of co-elution, and thus increasing the number of separated compounds [4].

A great increase in the number of separated compounds can also be attained by using comprehensive two-dimensional GC-MS (GC × GC-MS). In GC × GC analyses, a dedicated transfer device (a cryogenic modulator in the majority of cases) is used to first cut, and then transfer fractions of effluent from a first analytical column (usually a conventional column) onto a second one (usually a short micro-bore column segment (1–2 m)) with a different stationary phase. Such a transfer (or modulation) process occurs sequentially, and in a continuous manner, throughout the analysis. The superiority of GC × GC, over conventional GC, is due to the: (I) enhanced selectivity; (II) increased separation power; (III) high sensitivity due to analyte re-concentration (if cryogenic modulation is used); (IV) pattern formation of homologous series of compounds (e.g., alkanes, fatty acid methyl esters, etc.), enhancing the reliability of identification. Comprehensive 2D GC was first introduced in 1991 [5], can now be considered as a well-known technology [6], and has been used both for the analysis of non-citrus and citrus essential oils [7,8].

With the aim of exploiting the benefits of both LC and GC × GC (with single quadrupole (Q) MS), in previous off-line research the two technologies were combined (LC//GC × GC-QMS) and used for the highly-detailed qualitative analysis of sweet orange and bergamot essential oils [9]. Later studies were focused on a highly specific albeit minor chemical class (sesquiterpene hydrocarbons) of lemon, bergamot, sweet orange, clementine, bitter orange, mandarin (green, yellow, red), pink grapefruit, and lime (Key A, Key B, and Persian) essential oils [10], and on the oxygenated constituents of green, yellow, and red mandarin oils [11].

In the present research, LC//GC × GC-QMS was used for the highly detailed qualitative profiling of the entire volatile fraction of Key A, Key B, and Persian lime oils. The scope of the study is to demonstrate and confirm the analytical power and potential of such a technique, in this case applied to lime essential oil. For such a reason, the research involved one of each type of lime essential oil.

## 2. Experimental

### 2.1. Samples and Sample Preparation

A C_7_-C_30_ *n*-alkane series was kindly provided by Merck Life Science (Merck KGaA, Darmstadt, Germany) for the calculation of LRI values.

Three genuine cold-pressed samples of lime (Key A, Key B, and Persian) oils were provided by Citrojugo S.A. de C.V. Tecomán (Colima, Mexico). Prior to LC analyses, the oils were diluted 1:2 (*v*/*v*) in hexane.

### 2.2. LC Pre-Separation

LC pre-separations were performed on the lime essential oils using the Shimadzu 5D Ultra-e system (Kyoto, Japan) consisting of:

(1) An LC system, equipped with a CBM-20A communication bus module, two LC-30AD dual-plunger parallel-flow pumps, a DGU-20A online degasser, an SPD-M20A photodiode array detector, a CTO-20A column oven, and an SIL-30AC autosampler. Data were acquired by the LC solution v.5.92 software (Shimadzu).

(2) An AOC-5000 auto injector equipped with a dedicated dual side-port syringe, employed as a transfer device (not used in the present investigation). LC fractions were collected by disconnecting the transfer line (linking the outlet of the LC detector to the syringe) from the syringe side.

LC conditions: a 100 × 3 mm ID × 5 µm d_p_ silica column (SUPELCOSIL LC-Si, Merck Life Science) was operated under the following gradient conditions (flow: 0.35 mL min^−1^): 0–4.5 min (100% hexane); from 4.5 to 6.0 min 100% MTBE (until the end of the analysis). Injection volume: 20 µL.

LC fractions: hydrocarbons were collected from 1.5 to 3 min (525 µL); oxygenated compounds were collected from 7.3 to 14 min (2345 µL).

Prior to GC × GC-QMS injection, the fractions were reduced to a volume of 100 µL (under a gentle stream of nitrogen).

### 2.3. GC × GC-QMS Analysis

All GC × GC-QMS applications were carried out on system consisting of a GC2010 gas chromatograph and a QP2010 Ultra quadrupole mass spectrometer (Shimadzu).

The primary column, an SLB-5 ms 30 m × 0.25 mm ID × 0.25 μm d_f_ column (Merck Life Science), was connected to an uncoated capillary segment (1.5 m × 0.18 mm ID, used to create a double-loop), using an SGE SilTite mini-union (Trajan, Ringwood, Victoria, Australia). The uncoated capillary was then connected to a segment of Supelcowax-10 (100% polyethylene glycol) 1.0 m × 0.10 mm ID × 0.10 μm d_f_ column (Merck Life Science), using another union (Trajan). Modulation was carried out every 5 s by using a loop-type modulator (under license from Zoex Corporation, Houston, TX, USA). The duration of the hot pulse (400 °C) was 400 ms.

GC oven temperature program: 50 °C to 250 °C at 3 °C min^−1^. Carrier gas, helium, was supplied at an initial pressure of 173.5 kPa (constant linear velocity). Injection temperature: 250 °C.

Injection mode and volume for monoterpene hydrocarbons: split (1:150), 0.4 μL.

Injection mode and volume for sesquiterpene hydrocarbons: split (1:20), 1.0 μL.

Injection mode and volume for oxygenated compounds: split (1:20), 1.0 μL.

Mass spectrometry parameters: the samples were analyzed in the scan mode using a mass range of 40–360 m/z; spectra generation frequency: 33 Hz; interface and ion source temperatures were 250 °C and 200 °C, respectively. MS ionization mode: electron ionization.

Data were collected by GCMS Solution v.4.45 software (Shimadzu, Kyoto, Japan); bidimensional visualization was carried out using ChromSquare v.2.3 software (Shimadzu). The MS database employed was the FFNSC 3.0 (Shimadzu).

## 3. Results

As performed in previous research [9], peak identification was carried out through the combined use of MS database spectral searching and LRI information (comparison between the MS database and experimental LRI values). Three levels of identification were defined: level I—a similarity match ≥90% and an experimental LRI value within a ± 5 LRI tolerance window, with respect to the database result; level II—either a similarity match ≥90%, or an experimental LRI value within a ± 5 LRI tolerance window, with respect to the database result (a compound identified in such a manner cannot be characterized by a similarity match <80%, or an experimental LRI value outside a ± 10 LRI tolerance range); level III—a similarity match >75% and an experimental LRI value within a ± 15 LRI tolerance window, with respect to the database result. It must be emphasized that pure standard compounds were not used in the present research to confirm peak identity. However, the combined use of LRI data and MS information is nowadays accepted for the identification of essential oil constituents [12]. Finally, the main scope of the research was to demonstrate the power of the off-line four-dimensional (4D) method for this type of food sample.

**Figure 1:**
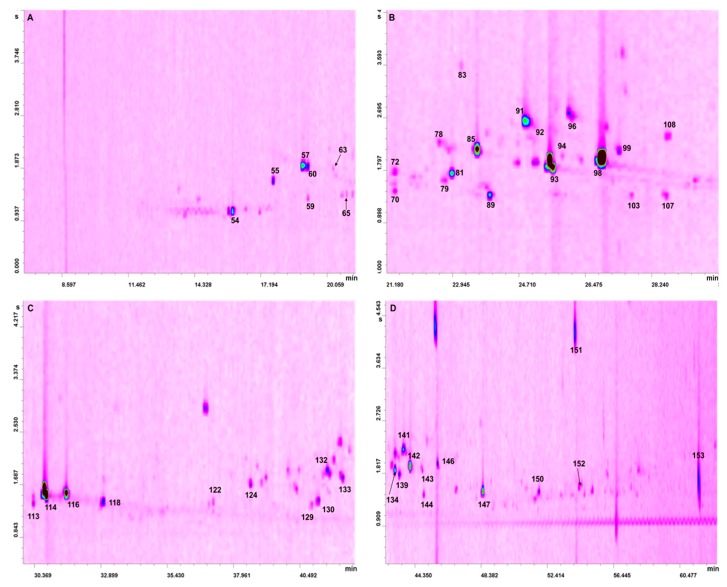
Four GC × GC-QMS chromatogram expansions (**A**–**D**) relative to the analysis of the oxygenated fraction of Persian lime oil (refer to Table 2 for peak identification).

After the LC pre-separation step, the two fractions (hydrocarbons and oxygenates) were reduced in volume (to 100 µL) and then subjected to three GC × GC-QMS analyses; the hydrocarbon fraction was analyzed twice, for the monoterpene (M) and sesquiterpene (S) hydrocarbons. For the latter compounds, present in lower quantities compared to the M hydrocarbons, a higher sample volume and lower split ratio were used. Fifty hydrocarbons were identified, considering the three oils: 46, 47, and 47 hydrocarbons in the Key A, Key B, and Persian lime oils, respectively, as shown in Table 1. With regard to the oxygenated compounds, an overall number of 103 constituents were identified: 77, 82, and 48 compounds in the Key A, Key B, and Persian lime oils, respectively, as shown in Table 2. The GC × GC-QMS chromatogram of the oxygenated fraction of the Persian lime oil is shown in four expansions in Figure 1A–D. As can be seen, more than half of the detected peaks in Figure 1A–D were not assigned.

Considering both the hydrocarbons and oxygenates, a total number of 153 constituents were identified in the three oils: 123, 129, and 95 compounds in the Key A, Key B, and Persian lime oils, respectively, as shown in Table 1 and Table 2.

## 4. Discussion

**Figure 2:**
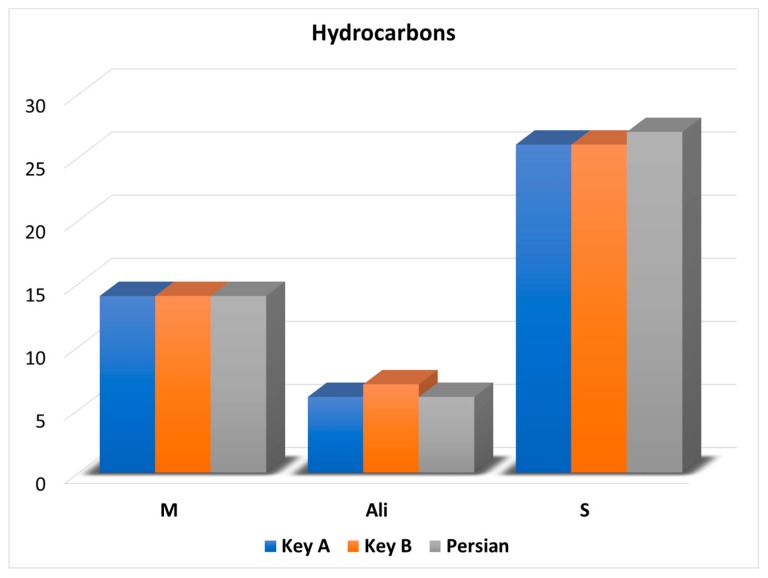
Graph illustrating the number and chemical class of the hydrocarbons identified in the three lime oil samples.

The off-line combination of HPLC and GC × GC–QMS, and its application to the detailed qualitative analysis of lime Essential oils (Eos), gave origin to compound-rich chromatograms, due to the possibility of concentrating the two pre-separated fractions (hydrocarbon and oxygenated compounds), and the two fundamental GC × GC characteristics, namely, the enhanced separation power and sensitivity. As mentioned previously, fifty hydrocarbons were identified with the distribution of M, S, and aliphatic hydrocarbons illustrated in the graph reported in Figure 2.

As can be observed, also in Table 1, the hydrocarbon profiles in the three types of lime oils were very similar. Considering the Key A oil, a number of compounds corresponding to 36, 9, and 1 were identified at levels I, II, and III, respectively; with regard to the Key B oil, a number of compounds corresponding to 32, 14, and 1 were identified at levels I, II, and III, respectively; finally, in the Persian oil, 38 and 9 compounds were identified at levels I and II, respectively. It is noteworthy that the LRI values were calculated by considering the total retention time (sum of the first and second dimension retention times) of the most intense modulated peak of both the alkanes and the lime oil hydrocarbons. Furthermore, the MS database LRI values were derived from analyses performed on the same (low polarity) column, as that used in the first analytical dimension. The retention of both the alkanes and the lime oil hydrocarbons, on the short medium-polarity (100% polyethylene glycol) second dimension, was negligible; for such a reason, there was a general good agreement between experimental and database LRI values.

Six hydrocarbons (all aliphatic) reported in Table 1, to the best of the present authors’ knowledge, have not been previously reported in the literature (an in-depth investigation was carried out) in a cold-extracted lime oil. Furthermore, γ-elemene (a sesquiterpene) was found for the first time in Persian oil, even though it has been reported in Key A and B oils [2,10]. Five hydrocarbons were found in both types of Key oils (undecane, tetradecane, pentadecane, hexadecane, heptadecane), while six (tetradec-1-ene, tetradecane, γ-elemene, pentadecane, hexadecane, heptadecane) were present in the Persian oil.

**Figure 3:**
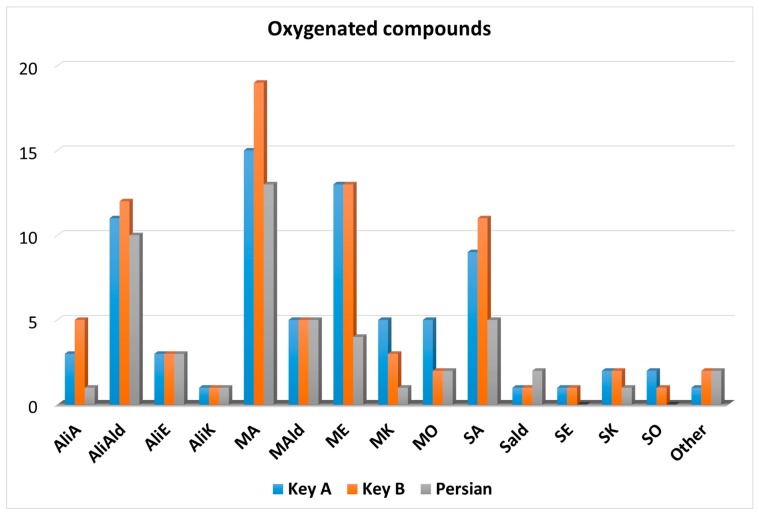
Graph illustrating the number and chemical class of the oxygenated compounds identified in the three lime oil samples.

The chemical class distribution of the 103 oxygenated compounds identified in the lime oils is illustrated in the graph shown in Figure 3.

The number of identified compounds was higher in the Key oils compared to the Persian one. Considering the Key A oil, a number of compounds corresponding to 46, 54, and 23 were identified at levels I, II, and III, respectively; with regard to the Key B oil, a number of compounds corresponding to 36, 73, and 20 were identified at levels I, II, and III, respectively; finally, in the Persian oil, 41, 46, and 8 compounds were identified at levels I, II, and III, respectively. Compared to the hydrocarbons, and in percentage terms, many more compounds were identified at levels II and III. Such an occurrence was, in part, due to the strong interaction of specific oxygenated compounds (e.g., alcohols) on the second dimension column, causing an increased divergence between the experimental and database LRIs.

After an in-depth investigation in the literature, no information was found on 65 compounds present in Table 2 and related to cold-pressed lime oil. Additionally, no previous description was found for the presence of tridecanal in Key B oil, even though it was identified in all the three oils [2]. Finally, (*E*)-nerolidol (a sesquiterpene alcohol) and dodecyl acetate were found in all the three oils, even though they have not been previously related to Persian lime oil [2].

To conclude, the applied LC//GC × GC-QMS method has enabled the in-depth elucidation of the chemical profile of three types of cold-pressed lime essential oils. The proposed method allows the formation of highly informative and ordered elution patterns that can be exploited for the creation of a fingerprint database as a support for quality assurance.

To the best of the authors’ knowledge, many volatiles are here related to such samples for the first time. It cannot obviously be excluded that, in cases, peak identification may not be correct (especially for level III identifications), and that compounds present in the literature related to cold-pressed lime oil have been missed. Even so, the 4D method herein proposed is a powerful analytical not only for citrus (and non-citrus) essential oil analysis, but also in other areas of food research. For example, the 4D technology has been used for the determination of mineral oil contamination in baby foods [13].

## Figures and Tables

**Table 1:** Hydrocarbons identified in the three types of cold-pressed lime essential oils, along with experimental and database linear retention index (LRI) values (exp./data LRI).

Peak	Hydrocarbon	Exp./Data LRI	Identification LEVEL			
			Key A	Key B	Persian	Class
1	Nonane ^a,b^	902/900	-	I	-	Ali
2	*α*-Thujene ^c^	927/927	I	I	I	M
3	*α*-Pinene ^c^	933/933	I	I	I	M
4	Camphene ^c^	953/953	I	I	I	M
5	Sabinene ^c^	973/972	I	I	I	M
6	*β*-Pinene ^c^	980/978	I	I	I	M
7	Myrcene ^c^	988/991	I	I	I	M
8	*α*-Phellandrene ^c^	1009/1007	I	I	I	M
9	*α*-Terpinene ^c^	1018/1018	I	I	I	M
10	*p*-Cymene ^c^	1025/1025	I	I	I	M
11	Limonene ^c^	1030/1030	I	I	I	M
12	β-Phellandrene	1045/1031	II ^c^	II ^d^	II ^c^	M
13	(E)-β-Ocimene ^c^	1047/1046	I	I	I	M
14	γ-Terpinene ^c^	1059/1058	I	I	I	M
15	Terpinolene ^c^	1087/1086	I	I	I	M
16	Undecane ^e^	1100/1100	II	II	-	Ali
17	Tridecane	1299/1300	I ^c^	I ^c^	I ^a^	Ali
18	*δ*-Elemene ^c^	1336/1335	I	I	I	S
19	*α*-Cubebene ^a^	1353/1349	II	II	-	S
20	*α*-Copaene ^a^	1381/1375	II	II	II	S
21	*β*-Elemene ^c^	1387/1390	I	I	I	S
22	Tetradec-1-ene ^e,b^	1391/1392	-	-	II	Ali
23	Tetradecane ^e^	1398/1400	I	II	I	Ali
24	(*Z*)-*α*-Bergamotene ^a^	1416/1416	I	I	I	S
25	*α*-Santalene ^a^	1422/1418	I	II	I	S
26	(*E*)-Caryophyllene ^c^	1426/1424	I	I	I	S
27	*γ*-Elemene	1434/1432	I ^c^	I ^c^	I ^e^	S
28	(*E*)-*α*-Bergamotene	1436/1432	I ^a^	I ^a^	I ^c^	S
29	*α*-Himachalene ^a^	1440/1449	I	II	I	S
30	(*E*)-*β*-Farnesene ^c^	1452/1452	I	I	I	S
31	*α*-Humulene ^c^	1462/1454	II	II	II	S
32	Sesquisabinene ^a^	1456/1455	I	II	I	S
33	*β*-Santalene ^c,b^	1464/1459	-	-	II	S
34	*β*-Chamigrene ^a^	1476/1479	II	II	II	S
35	*γ*-Curcumene ^a^	1481/1482	I	I	I	S
36	*α*-Curcumene ^a^	1483/1480	-	-	II	S
37	Germacrene D ^a^	1487/1480	II	II	II	S
38	(*E*)-*β*-Bergamotene ^a^	1488/1483	II	II	I	S
39	Valencene ^a^	1490/1492	I	I	I	S
40	*β*-Selinene	1497/1492	I ^c^	I ^c^	I ^a^	S
41	Pentadecane ^e^	1498/1500	III	III	I	Ali
42	(*Z*)-*α*-Bisabolene ^a^	1503/1503	I	I	I	S
43	(*E*, *E*) -*α*-Farnesene ^c^	1505/1504	I	I	I	S
44	*β*-Bisabolene ^c^	1509/1508	I	I	I	S
45	(*Z*)-*γ*-Bisabolene ^a^	1511/1515	I	I	I	S
46	(*E*)-*γ*-Bisabolene ^a^	1530/1528	I	II	I	S
47	(*E*)-*α*-Bisabolene ^a^	1541/1540	I	I	I	S
48	Germacrene B ^c^	1556/1557	I	I	II	S
49	Hexadecane ^e^	1598/1600	II	II	I	Ali
50	Heptadecane ^e^	1699/1700	I	I	I	Ali

Abbreviations: M: monoterpene; Ali: aliphatic; S: sesquiterpene. ^a^ Compound not yet identified in cold-extracted laboratory oils [2,10]. ^b^ Compound identified in only one of the samples. ^c^ Compound identified previously in industrially cold-extracted lime oils and cold-extracted laboratory oils, reported since 1980 [2,10]. ^d^ Compound identified previously only in cold-extracted laboratory oils [2,10]. ^e^ Compound, to the best of the authors’ knowledge, identified for the first time in an industrially cold-extracted lime oil.

**Table 2:** Oxygenated compounds identified in the three types of cold-pressed lime essential oils, along with experimental and database LRI values (exp./data LRI).

Peak	Oxygenated Compound	Exp./Data LRI	Identification Level			
			Key A	Key B	Persian	Class
51	Pinacol ^a^	862/858	III	III	-	AliA
52	6-methyl-5-hepten-2-one ^b^	985/986	I	II	-	AliK
53	Octanal ^c^	1006/1006	I	I	-	AliAld
54	Eucalyptol ^a^	1036/1032	II	-	II	MA
55	(*Z*)-Sabinene hydrate ^c^	1074/1069	II	II	II	MA
56	Octanol ^c^	1074/1076	II	-	-	AliA
57	Linalool ^c^	1101/1101	I	I	I	MA
58	(*E*)-Sabinene hydrate ^c^	1105/1099	II	-	-	MA
59	Nonanal ^c^	1106/1107	II	II	II	AliAld
60	(*E*)-Pinene hydrate ^a^	1111/1121	-	II	II	MA
61	Endo-fenchol ^b,d^	1126/1119	-	II	-	MA
62	(*E*)-*p*-Mentha-2,8-dien-1-ol ^a^	1128/1122	II	III	-	MA
63	(*E*)-*p*-Menth-2-en-1ol	1129/1139	III ^b^	II ^b^	II ^c^	MA
64	(*3E,6Z*) -Nonadienol ^a,d^	1141/1152	-	III	-	AliA
65	(*Z*)-Limonene oxide ^c^	1142/1134	II	-	II	MO
66	(*E*)-Limonene oxide ^c^	1142/1138	II	-	-	MO
67	(*Z*)-*p*-Mentha-2,8-dien-1-ol ^a,d^	1142/1138	-	II	-	MA
68	(*E*)-Myroxide ^a^	1147/1141	III	-	-	MO
69	(*E*)-Pinocarveol ^b^	1147/1141	-	II	II	MA
70	Citronellal ^c^	1154/1152	II	II	II	MAld
71	Camphor ^a,d^	1154/1149	-	III	-	MK
72	Isopulegol ^a^	1154/1149	III	II	II	MA
73	(*Z*)-Non-3-en-1-ol ^a,d^	1163/1153	-	III	-	AliA
74	Camphene hydrate ^a,d^	1163/1156	-	II	-	MA
75	Pinocarvone ^a^	1168/1164	II	II	-	MK
76	Non-(*2Z*)-enol ^a,d^	1171/1170	-	III	-	AliA
77	Rose furan oxide ^a^	1172/1169	II	II	-	MO
78	Borneol	1179/1173	II ^b^	II ^b^	II ^c^	MA
79	Isogeranial ^a^	1182/1179	III	II	II	MAld
80	(*Z*)-Pinocamphone ^b,d^	1182/1176	II	-	-	MK
81	Terpinen-4-ol ^c^	1186/1180	II	II	II	MA
82	(*Z*)-Pinocarveol ^a^	1188/1186	-	II	-	MA
83	*p*-Cymen-8-ol	1192/1189	II ^a^	II ^b^	III ^a^	MA
84	Non-(*6Z*)-enal ^a,d^	1196/1206	III	-	-	AliAld
85	*α*-Terpineol ^c^	1200/1195	I	I	I	MA
86	Dec-(*4Z*)-enal ^a,d^	1196/1196	-	II	-	AliAld
87	(*Z*)-Piperitol ^a^	1207/1198	III	II	-	MA
88	*neo*-Dihydro carveol ^a,d^	1203/1198	II	-	-	MA
89	Decanal ^c^	1207/1208	III	II	II	AliAld
90	(*E*)-Piperitol ^e,d^	1216/1208	-	II	-	MA
91	Nerol ^c^	1231/1229	I	II	II	MA
92	3,7-dimethyl-Oct-7-enol ^a,d^	1231/1228	-	-	III	AliA
93	Neral ^c^	1242/1238	I	II	I	MAld
94	Carvone ^a^	1249/1246	II	-	II	MK
95	Linalyl acetate ^a^	1250/1250	III	II	-	ME
96	Geraniol ^c^	1256/1255	II	II	II	MA
97	Piperitone ^b^	1260/1267	II	II	-	MK
98	Geranial ^c^	1272/1268	I	II	II	MAld
99	Perilla aldehyde ^c^	1282/1278	I	II	II	MAld
100	Dihydro-linalool acetate ^a,d^	1286/1275	-	II	-	ME
101	Dec-2-en-1-ol ^a,d^	1284/1270	III	-	-	AliA
102	*iso*-Isopulegyl acetate ^a^	1289/1286	II	II	-	ME
103	Thujyl acetate ^a^	1289/1298	-	II	II	ME
104	(*Z*)-Verbenyl acetate ^a^	1290/1278	III	II	-	ME
105	(*E*)-Pinocarvyl acetate ^a^	1298/1296	II	II	-	ME
106	Geranyl formate ^d,e^	1298/1300	II	-	-	ME
107	Undecanal ^c^	1307/1309	II	II	II	AliAld
108	Isoascaridole ^a^	1309/1306	II	II	II	MO
109	Deca-(*2E,4E*)-dienal ^a^	1321/1322	III	II	-	AliAld
110	Methyl geranate ^a^	1322/1326	II	II	-	ME
111	Myrtenyl acetate ^a^	1326/1326	II	II	-	ME
112	Citronellyl acetate ^c^	1349/1350	II	II	-	ME
113	*neo-iso*-Carvomenthyl acetate ^a,d^	1349/1350	-	-	II	ME
114	Neryl acetate ^c^	1359/1361	II	II	II	ME
115	(*E*)-Myrtanol acetate ^a,d^	1372/1387	II	-	-	ME
116	Geranyl acetate ^c^	1378/1380	I	II	II	ME
117	(*Z*)-Trimenal ^a,d^	1435/1424	-	III	-	AliAld
118	Dodecanal ^c^	1411/1410	II	II	II	AliAld
119	(*E*)-Nerone ^a,d^	1435/1440	III	-	-	MK
120	(*E*)-Trimenal ^a,d^	1435/1424	-	III	-	AliAld
121	Geranyl isobutyrate ^a^	1506/1507	III	III	-	ME
122	Tridecanal	1512/1516	II ^b^	II ^a^	II ^c^	AliAld
123	(*Z*)-Nerolidol ^a^	1544/1531	-	III	-	SA
124	(*E*)-Nerolidol	1551/1561	III ^e^	II ^e^	III ^a^	SA
125	Hedycaryol ^a^	1554/1544	II	II	-	SA
126	Longipinanol ^a^	1558/1572	III	III	-	SA
127	(*E*)-Sesquisabinene hydrate ^a^	1584/1576	II	II	-	SA
128	Caryophyllene oxide ^c^	1592/1587	III	II	-	SO
129	Dodecyl acetate	1607/1610	II ^e^	II ^c^	II ^a^	AliE
130	Tetradecanal ^c^	1614/1614	II	II	II	AliAld
131	Humulene epoxide II ^a,d^	1619/1613	III	-	-	SO
132	(*Z*)-Sesquilavandulol ^a^	1623/1610	III	III	III	SA
133	(*E*)-Sesquilavandulol ^a^	1639/1633	II	II	II	SA
134	(*E*)-Tetradec-2-enal ^a,d^	1668/1673	-	-	III	AliAld
135	(*Z*)-Nerolidyl acetate ^a,d^	1664/1665	II	-	-	SE
136	*epi-α*-Bisabolol ^a,d^	1664/1679	-	III	-	SA
137	Isobornyl isobutanoate-8-hydroxy ^a^	1668/1676	II	II	-	ME
138	*neo*-Intermedeol ^a^	1668/1661	II	II	-	SA
139	*β*-Bisabolol ^a^	1677/1677	II	II	II	SA
140	(*Z*)-Apritone ^a^	1688/1687	I	II	II	SK
141	*α*-Bisabolol ^c^	1693/1688	II	I	II	SA
142	(*E*)-Apritone ^a^	1713/1710	II	II	-	SK
143	(*2E,6Z*) - Farnesal ^a,d^	1713/1714	-	-	II	SAld
144	Tridec-2-en-1-ol acetate ^a^	1715/1705	III	III	III	AliE
145	Hernianin ^a,d^	1735/1720	-	III	-	Other
146	(*E, E*) -Farnesal ^a^	1739/1737	II	II	II	SAld
147	Hexadec-(*11Z*)-enal ^a^	1817/1808	II	II	II	AliAld
148	Farnesyl acetate ^a, d^	1832/1832	-	III	-	SE
149	Hexadec-(*11E*)-en-1-ol ^a,d^	1879/1869	-	III	-	AliA
150	Cyclohexadecanolide ^a^	1920/1935	III	III	III	AliE
151	Citropten ^a^	1991/1982	II	II	II	Other
152	Octadec-(*13Z*)-enal ^a^	1998/2010	III	III	III	AliAld
153	Isopimpinellin ^a, d^	2239/2239	-	-	II	Other

Abbreviations: Ali, aliphatic; K, ketone; Ald, aldehyde; E, ester; O, oxide; A, alcohol. ^a^ Compound, to the best of the authors’ knowledge, identified for the first time in an industrially cold-extracted lime oil. ^b^ Compound not yet identified in cold-extracted laboratory oils [2]. ^c^ Compound identified previously in industrially cold-extracted lime oils and cold-extracted laboratory oils, reported since 1980 [2]. ^d^ Compound identified in only one of the samples. ^e^ Compound identified previously only in cold-extracted laboratory oils [2].
